# (*E*)-1-(4-Fluoro­phen­yl)ethan-1-one semicarbazone

**DOI:** 10.1107/S1600536809022521

**Published:** 2009-06-20

**Authors:** Hoong-Kun Fun, Chin Sing Yeap, Mahesh Padaki, Shridhar Malladi, Arun M. Isloor

**Affiliations:** a X-ray Crystallography Unit, School of Physics, Universiti Sains Malaysia, 11800 USM, Penang, Malaysia; b Department of Chemistry, National Institute of Technology–Karnataka, Surathkal, Mangalore 575 025, India

## Abstract

In the title compound, C_9_H_10_FN_3_O, the semicarbazone group is nearly planar, with the maximum deviation of 0.044 (1) Å for one of the N atoms. The mean plane of semicarbazone group forms a dihedral angle of 30.94 (4)° with the benzene ring. The mol­ecules are linked into a supra­molecular chain by N—H⋯O hydrogen bonds formed along the *c* axis. The crystal structure is further stabilized by weak inter­molucular C—H⋯π inter­actions; the closest C⋯*Cg* contact is 3.6505 (11) Å.

## Related literature

For hydrogen-bond motifs, see: Bernstein *et al.* (1995 ▶). For applications of semicarbazone derivatives, see: Chandra & Gupta (2005 ▶); Jain *et al.* (2002 ▶); Pilgram (1978 ▶); Warren *et al.* (1977 ▶); Yogeeswari *et al.* (2004 ▶). For the preparation of the compound, see: Furniss *et al.* (1978 ▶). For related structures, see: Fun *et al.* (2009*a* ▶,*b* ▶). For the stability of the temperature controller used for the data collection, see: Cosier & Glazer (1986 ▶).
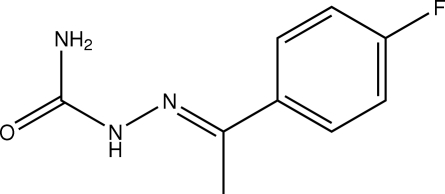


## Experimental

### 

#### Crystal data

C_9_H_10_FN_3_O
*M* *_r_* = 195.20
Monoclinic, 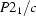

*a* = 18.8207 (3) Å
*b* = 6.6387 (1) Å
*c* = 7.3074 (1) Å
β = 95.887 (1)°
*V* = 908.21 (2) Å^3^
*Z* = 4
Mo *K*α radiation
μ = 0.11 mm^−1^
*T* = 100 K
0.30 × 0.10 × 0.08 mm

#### Data collection

Bruker SMART APEXII CCD area-detector diffractometer
Absorption correction: multi-scan (**SADABS**; Bruker, 2005 ▶) *T*_min_ = 0.876, *T*_max_ = 0.991
17523 measured reflections
3998 independent reflections
2766 reflections with *I* > 2σ(*I*)
*R*_int_ = 0.033

#### Refinement

*R*[*F* ^2^ > 2σ(*F* ^2^)] = 0.049
*wR*(*F* ^2^) = 0.151
*S* = 1.07
3998 reflections
167 parameters
H atoms treated by a mixture of independent and constrained refinement
Δρ_max_ = 0.51 e Å^−3^
Δρ_min_ = −0.30 e Å^−3^

### 

Data collection: *APEX2* (Bruker, 2005 ▶); cell refinement: *SAINT* (Bruker, 2005 ▶); data reduction: *SAINT*; program(s) used to solve structure: *SHELXTL* (Sheldrick, 2008 ▶); program(s) used to refine structure: *SHELXTL*; molecular graphics: *SHELXTL*; software used to prepare material for publication: *SHELXTL* and *PLATON* (Spek, 2009 ▶).

## Supplementary Material

Crystal structure: contains datablocks global, I. DOI: 10.1107/S1600536809022521/tk2477sup1.cif

Structure factors: contains datablocks I. DOI: 10.1107/S1600536809022521/tk2477Isup2.hkl

Additional supplementary materials:  crystallographic information; 3D view; checkCIF report

## Figures and Tables

**Table 1:** Hydrogen-bond geometry (Å, °)

*D*—H⋯*A*	*D*—H	H⋯*A*	*D*⋯*A*	*D*—H⋯*A*
N2—H1*N*2⋯O1^i^	0.96 (2)	1.94 (2)	2.8998 (11)	179.2 (18)
N3—H2*N*3⋯O1^ii^	0.82 (2)	2.07 (2)	2.8901 (12)	176 (2)
C2—H2*A*⋯*Cg*1^iii^	0.989 (17)	2.927 (18)	3.7250 (12)	138.5 (12)
C5—H5*A*⋯*Cg*1^iv^	0.976 (14)	2.825 (13)	3.6505 (11)	142.8 (10)

Symmetry codes: (i) 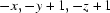; (ii) 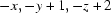; (iii) 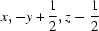; (iv) 
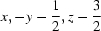
. *Cg*1 is the centroid of C1–C6 benzene ring.

**Fig. 1.**
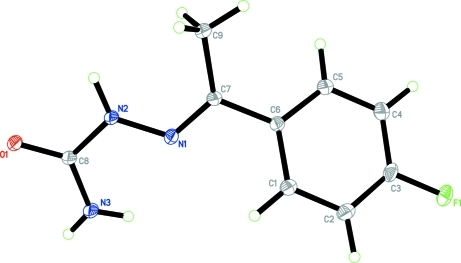
The molecular structure of (I) with atom labels and 50% probability ellipsoids for non-H atoms.

**Fig. 2.**
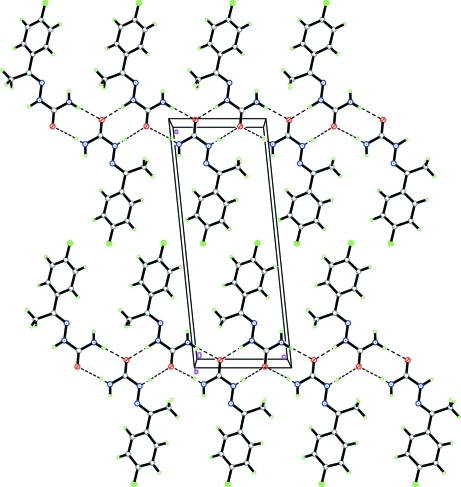
The crystal packing of (I), viewed down the b axis, showing the molecules are linked along the c axis. Hydrogen bonds are shown in as dashed lines.

## supplementary crystallographic information

### Comment

In organic chemistry, a semicarbazone is a derivative of an aldehyde or ketone formed by a condensation between a ketone or aldehyde and semicarbazide. Semicarbazones find a large number of applications in the field of synthetic chemistry, such as in medicinal chemistry (Warren *et al.*, 1977), organometallics (Chandra & Gupta, 2005), polymers (Jain *et al.*, 2002), and herbicides (Pilgram, 1978). 4-Sulphamoylphenyl semicarbazones were found to possess anti-convulsant activity (Yogeeswari *et al.*, 2004). We hereby report the crystal structure of a semicarbazone of commercial importance, (I).

The bond lengths and angles for (I), Fig. 1, are comparable to those found in related structures (Fun *et al.*, 2009a, b). A maximum deviation of 0.044 (1) Å for atom N2 from the mean plane formed by atoms O1, N1, N2, N3, C6, C7, C8 and C9, indicates that the semicarbazone group is nearly planar. This mean plane makes a dihedral angle of 30.94 (4)° with the C1–C6 benzene ring. The molecules are linked into one-dimensional chains by intermolecular N—H···O hydrogen bonds along the *c* axis (Fig. 2); these hydrogen bonding interactions generate *R*_2_^2^(8) ring motifs (Bernstein *et al.*, 1995). The crystal structure is stabilized by weak intermolecular C—H···π interactions (Table 1).

### Experimental

Semicarbazide hydrochloride (0.86 g, 7.70 mmol) and freshly recrystallized sodium acetate (0.77 g, 9.40 mmol) were dissolved in water (10 ml) following a literature procedure (Furniss *et al.*,  1978). The reaction mixture was stirred at room temperature for 10 minutes. To this, 4-fluoroacetophenone (1.00 g, 7.23 mmol) was added and the mixture was shaken well. A little alcohol was added to dissolve the turbidity. The mixture was shaken for a further 10 minutes and allowed to stand. The title compound (I) crystallizes on standing for 6 h. The  separated crystals were filtered, washed with cold water and recrystallized from ethanol. Yield: 1.34 g (95%). M.p. 485-486 K.

### Refinement

All hydrogen atoms were located from the difference Fourier map and refined freely, N—H = 0.820 (19) - 0.957 (19) Å and C–H = 0.945 (19) - 1.005 (18) Å.

### Crystal data

**Table:**

C_9_H_10_FN_3_O	*F*(000) = 408
*M**_r_* = 195.20	*D*_x_ = 1.428 Mg m^−^^3^
Monoclinic, *P*2_1_/*c*	Mo *K*α radiation, λ = 0.71073 Å
Hall symbol: -P 2ybc	Cell parameters from 5018 reflections
*a* = 18.8207 (3) Å	θ = 4.2–38.8°
*b* = 6.6387 (1) Å	µ = 0.11 mm^−^^1^
*c* = 7.3074 (1) Å	*T* = 100 K
β = 95.887 (1)°	Needle, colourless
*V* = 908.21 (2) Å^3^	0.30 × 0.10 × 0.08 mm
*Z* = 4	

### Data collection

**Table:**

Bruker SMART APEXII CCD area-detector diffractometer	3998 independent reflections
Radiation source: fine-focus sealed tube	2766 reflections with *I* > 2σ(*I*)
graphite	*R*_int_ = 0.033
φ and ω scans	θ_max_ = 35.0°, θ_min_ = 1.1°
Absorption correction: multi-scan (*SADABS*; Bruker, 2005)	*h* = −29→30
*T*_min_ = 0.876, *T*_max_ = 0.991	*k* = −10→10
17523 measured reflections	*l* = −7→11

### Refinement

**Table:**

Refinement on *F*^2^	Primary atom site location: structure-invariant direct methods
Least-squares matrix: full	Secondary atom site location: difference Fourier map
*R*[*F*^2^ > 2σ(*F*^2^)] = 0.049	Hydrogen site location: inferred from neighbouring sites
*wR*(*F*^2^) = 0.151	H atoms treated by a mixture of independent and constrained refinement
*S* = 1.07	*w* = 1/[σ^2^(*F*_o_^2^) + (0.078*P*)^2^ + 0.1557*P*] where *P* = (*F*_o_^2^ + 2*F*_c_^2^)/3
3998 reflections	(Δ/σ)_max_ < 0.001
167 parameters	Δρ_max_ = 0.51 e Å^−^^3^
0 restraints	Δρ_min_ = −0.30 e Å^−^^3^

### Special details

**Table:**

Experimental. The crystal was placed in the cold stream of an Oxford Cyrosystems Cobra open-flow nitrogen cryostat (Cosier & Glazer, 1986) operating at 100.0 (1)K.
Geometry. All esds (except the esd in the dihedral angle between two l.s. planes) are estimated using the full covariance matrix. The cell esds are taken into account individually in the estimation of esds in distances, angles and torsion angles; correlations between esds in cell parameters are only used when they are defined by crystal symmetry. An approximate (isotropic) treatment of cell esds is used for estimating esds involving l.s. planes.
Refinement. Refinement of F^2^ against ALL reflections. The weighted R-factor wR and goodness of fit S are based on F^2^, conventional R-factors R are based on F, with F set to zero for negative F^2^. The threshold expression of F^2^ > 2sigma(F^2^) is used only for calculating R-factors(gt) etc. and is not relevant to the choice of reflections for refinement. R-factors based on F^2^ are statistically about twice as large as those based on F, and R- factors based on ALL data will be even larger.

### Fractional atomic coordinates and isotropic or equivalent isotropic displacement parameters (Å^2^)

**Table:**

	*x*	*y*	*z*	*U*_iso_*/*U*_eq_	
F1	0.50077 (4)	0.48694 (11)	0.79001 (10)	0.02274 (17)	
O1	−0.01336 (4)	0.50372 (12)	0.73484 (10)	0.01547 (16)	
N1	0.16753 (5)	0.50702 (12)	0.66798 (11)	0.01257 (16)	
N2	0.09495 (5)	0.50670 (13)	0.62389 (11)	0.01339 (17)	
N3	0.08724 (5)	0.50728 (15)	0.93755 (12)	0.01734 (19)	
C1	0.31185 (5)	0.59520 (16)	0.76477 (14)	0.01595 (19)	
C2	0.38374 (6)	0.59368 (17)	0.82935 (14)	0.0178 (2)	
C3	0.43028 (5)	0.49203 (16)	0.72738 (15)	0.01591 (19)	
C4	0.40812 (6)	0.39453 (17)	0.56461 (14)	0.0177 (2)	
C5	0.33579 (5)	0.39948 (17)	0.50051 (13)	0.01569 (19)	
C6	0.28652 (5)	0.49812 (14)	0.60026 (13)	0.01153 (17)	
C7	0.20888 (5)	0.49673 (14)	0.53803 (13)	0.01158 (17)	
C8	0.05269 (5)	0.50588 (15)	0.76711 (13)	0.01249 (18)	
C9	0.18228 (6)	0.48336 (16)	0.33718 (13)	0.01471 (19)	
H1A	0.2783 (8)	0.667 (2)	0.8362 (19)	0.023 (4)*	
H2A	0.4017 (8)	0.666 (3)	0.943 (2)	0.031 (4)*	
H4A	0.4422 (7)	0.324 (2)	0.4966 (19)	0.021 (3)*	
H5A	0.3214 (7)	0.328 (2)	0.3860 (19)	0.017 (3)*	
H9A	0.1565 (9)	0.602 (3)	0.303 (2)	0.035 (4)*	
H9B	0.1536 (9)	0.364 (3)	0.317 (2)	0.037 (5)*	
H9C	0.2216 (10)	0.480 (2)	0.254 (2)	0.031 (5)*	
H1N2	0.0681 (10)	0.502 (2)	0.505 (3)	0.029 (4)*	
H1N3	0.1329 (10)	0.506 (2)	0.947 (2)	0.025 (4)*	
H2N3	0.0643 (10)	0.505 (2)	1.027 (3)	0.030 (5)*	

### Atomic displacement parameters (Å^2^)

**Table:**

	*U*^11^	*U*^22^	*U*^33^	*U*^12^	*U*^13^	*U*^23^
F1	0.0096 (3)	0.0346 (4)	0.0230 (3)	0.0012 (2)	−0.0031 (2)	−0.0014 (3)
O1	0.0103 (3)	0.0256 (4)	0.0103 (3)	−0.0002 (3)	0.0004 (2)	−0.0005 (3)
N1	0.0098 (3)	0.0167 (4)	0.0110 (3)	0.0002 (3)	0.0002 (3)	0.0005 (3)
N2	0.0101 (3)	0.0220 (4)	0.0080 (3)	−0.0003 (3)	0.0004 (3)	0.0000 (3)
N3	0.0120 (4)	0.0317 (5)	0.0082 (3)	−0.0002 (3)	0.0003 (3)	−0.0006 (3)
C1	0.0137 (4)	0.0188 (5)	0.0150 (4)	0.0013 (4)	−0.0002 (3)	−0.0034 (3)
C2	0.0152 (4)	0.0214 (5)	0.0161 (4)	0.0001 (4)	−0.0022 (3)	−0.0036 (4)
C3	0.0097 (4)	0.0205 (5)	0.0169 (4)	0.0002 (3)	−0.0017 (3)	0.0026 (3)
C4	0.0131 (4)	0.0230 (5)	0.0173 (4)	0.0029 (4)	0.0021 (3)	−0.0016 (4)
C5	0.0134 (4)	0.0202 (5)	0.0133 (4)	0.0012 (3)	0.0006 (3)	−0.0024 (3)
C6	0.0107 (4)	0.0134 (4)	0.0103 (4)	0.0005 (3)	0.0002 (3)	0.0007 (3)
C7	0.0116 (4)	0.0128 (4)	0.0102 (4)	0.0005 (3)	0.0003 (3)	−0.0006 (3)
C8	0.0114 (4)	0.0167 (4)	0.0094 (4)	0.0000 (3)	0.0009 (3)	−0.0005 (3)
C9	0.0122 (4)	0.0211 (5)	0.0105 (4)	−0.0010 (4)	−0.0003 (3)	−0.0006 (3)

### Geometric parameters (Å, °)

**Table:**

F1—C3	1.3587 (12)	C2—C3	1.3826 (15)
O1—C8	1.2413 (12)	C2—H2A	0.989 (17)
N1—C7	1.2900 (13)	C3—C4	1.3805 (15)
N1—N2	1.3709 (12)	C4—C5	1.3939 (14)
N2—C8	1.3779 (13)	C4—H4A	0.971 (14)
N2—H1N2	0.957 (19)	C5—C6	1.3995 (14)
N3—C8	1.3446 (13)	C5—H5A	0.976 (14)
N3—H1N3	0.856 (18)	C6—C7	1.4851 (13)
N3—H2N3	0.820 (19)	C7—C9	1.5040 (13)
C1—C2	1.3866 (14)	C9—H9A	0.945 (19)
C1—C6	1.4037 (14)	C9—H9B	0.964 (18)
C1—H1A	0.983 (14)	C9—H9C	1.005 (18)
			
C7—N1—N2	119.27 (8)	C4—C5—C6	120.83 (9)
N1—N2—C8	117.41 (8)	C4—C5—H5A	116.8 (8)
N1—N2—H1N2	129.3 (10)	C6—C5—H5A	122.3 (8)
C8—N2—H1N2	113.3 (10)	C5—C6—C1	118.42 (9)
C8—N3—H1N3	117.6 (12)	C5—C6—C7	121.42 (8)
C8—N3—H2N3	119.6 (13)	C1—C6—C7	120.14 (8)
H1N3—N3—H2N3	122.7 (18)	N1—C7—C6	115.04 (8)
C2—C1—C6	121.47 (9)	N1—C7—C9	123.77 (9)
C2—C1—H1A	118.7 (8)	C6—C7—C9	121.19 (8)
C6—C1—H1A	119.8 (8)	O1—C8—N3	123.76 (9)
C3—C2—C1	118.04 (9)	O1—C8—N2	120.04 (9)
C3—C2—H2A	120.6 (9)	N3—C8—N2	116.20 (9)
C1—C2—H2A	121.4 (9)	C7—C9—H9A	108.5 (11)
F1—C3—C4	118.48 (9)	C7—C9—H9B	108.7 (10)
F1—C3—C2	118.77 (9)	H9A—C9—H9B	112.6 (16)
C4—C3—C2	122.75 (10)	C7—C9—H9C	113.6 (11)
C3—C4—C5	118.48 (10)	H9A—C9—H9C	104.5 (14)
C3—C4—H4A	120.7 (8)	H9B—C9—H9C	109.0 (14)
C5—C4—H4A	120.8 (8)		
			
C7—N1—N2—C8	176.26 (9)	C2—C1—C6—C7	177.96 (9)
C6—C1—C2—C3	−0.43 (16)	N2—N1—C7—C6	179.99 (8)
C1—C2—C3—F1	−179.17 (10)	N2—N1—C7—C9	−0.23 (14)
C1—C2—C3—C4	0.56 (17)	C5—C6—C7—N1	150.01 (10)
F1—C3—C4—C5	179.90 (9)	C1—C6—C7—N1	−28.31 (13)
C2—C3—C4—C5	0.17 (17)	C5—C6—C7—C9	−29.77 (14)
C3—C4—C5—C6	−1.05 (16)	C1—C6—C7—C9	151.91 (10)
C4—C5—C6—C1	1.16 (15)	N1—N2—C8—O1	−179.35 (9)
C4—C5—C6—C7	−177.18 (9)	N1—N2—C8—N3	0.57 (13)
C2—C1—C6—C5	−0.41 (15)		

### Hydrogen-bond geometry (Å, °)

**Table:**

*D*—H···*A*	*D*—H	H···*A*	*D*···*A*	*D*—H···*A*
N2—H1N2···O1^i^	0.96 (2)	1.94 (2)	2.8998 (11)	179.2 (18)
N3—H2N3···O1^ii^	0.82 (2)	2.07 (2)	2.8901 (12)	176 (2)
C2—H2A···Cg1^iii^	0.989 (17)	2.927 (18)	3.7250 (12)	138.5 (12)
C5—H5A···Cg1^iv^	0.976 (14)	2.825 (13)	3.6505 (11)	142.8 (10)

Symmetry codes: (i) −*x*, −*y*+1, −*z*+1; (ii) −*x*, −*y*+1, −*z*+2; (iii) *x*, −*y*+1/2, *z*−1/2; (iv) *x*, −*y*−1/2, *z*−3/2.
